# First Report on Detection and Molecular Characterization of Adenoviruses in the Small Indian Mongoose (*Urva auropunctata*)

**DOI:** 10.3390/v13112194

**Published:** 2021-10-30

**Authors:** Kerry Gainor, Anne A. M. J. Becker, Yashpal S. Malik, Souvik Ghosh

**Affiliations:** 1 Department of Biomedical Sciences, Ross University School of Veterinary Medicine, P.O. Box 334, 00265 Basseterre, St. Kitts and Nevis, West Indies; KerryGainor@students.rossu.edu (K.G.); ABecker@rossvet.edu.kn (A.A.M.J.B.); 2 College of Animal Biotechnology, Guru Angad Dev Veterinary and Animal Science University, 141004 Ludhiana, India; malikyps@gmail.com

**Keywords:** adenovirus, small Indian mongoose, atadenovirus, mastadenovirus, DNA-dependent DNA polymerase (pol)

## Abstract

Using a broad-range nested PCR assay targeting the DNA-dependent DNA polymerase (*pol*) gene, we detected adenoviruses in 17 (20.48%) out of 83 fecal samples from small Indian mongooses (*Urva auropunctata*) on the Caribbean island of St. Kitts. All 17 PCR amplicons were sequenced for the partial *pol* gene (~300 bp, hereafter referred to as Mon sequences). Fourteen of the 17 Mon sequences shared maximum homology (98.3–99.6% and 97–98.9% nucleotide (nt) and deduced amino acid (aa) sequence identities, respectively) with that of bovine adenovirus-6 (species *Bovine atadenovirus E*). Mongoose-associated adenovirus Mon-39 was most closely related (absolute nt and deduced aa identities) to an atadenovirus from a tropical screech owl. Mon-66 shared maximum nt and deduced aa identities of 69% and 71.4% with those of atadenoviruses from a spur-thighed tortoise and a brown anole lizard, respectively. Phylogenetically, Mon-39 and Mon-66 clustered within clades that were predominated by atadenoviruses from reptiles, indicating a reptilian origin of these viruses. Only a single mongoose-associated adenovirus, Mon-34, was related to the genus *Mastadenovirus*. However, phylogenetically, Mon-34 formed an isolated branch, distinct from other mastadenoviruses. Since the fecal samples were collected from apparently healthy mongooses, we could not determine whether the mongoose-associated adenoviruses infected the host. On the other hand, the phylogenetic clustering patterns of the mongoose-associated atadenoviruses pointed more towards a dietary origin of these viruses. Although the present study was based on partial pol sequences (~90 aa), sequence identities and phylogenetic analysis suggested that Mon-34, Mon-39, and Mon-66 might represent novel adenoviruses. To our knowledge, this is the first report on the detection and molecular characterization of adenoviruses from the mongoose.

## 1. Introduction

Adenoviruses, members of the family *Adenoviridae*, are double-stranded DNA viruses that have been detected in a wide variety of vertebrates [1,2]. Although most adenovirus infections are asymptomatic or cause mild disease in immunocompetent hosts, certain adenoviruses have been associated with acute clinical conditions in humans and animals [1,3]. To date, the International Committee on Taxonomy of Viruses (ICTV) has formally recognized 6 genera and 86 species within the family *Adenoviridae* (https://talk.ictvonline.org/ictv-reports/ictv_9th_report/dsdna-viruses-2011/w/dsdna_viruses/93/adenoviridae, accessed on 10 October 2021). The genus *Mastadenovirus* and *Aviadenovius* consists of adenoviruses of mammalian and avian host origin, respectively [1,2]. Viruses belonging to the genus *Atadenovirus* have been reported in a wide range of animals, including birds, marsupials, reptiles (Order *Squamata*), ruminants, and a spur-thighed tortoise [2]. Members of the genus *Siadenovirus* have been detected in birds, a frog, and a few tortoise species [2]. The genus *Ichtadenovirus* is composed of a single adenovirus species from a fish (White sturgeon) [2]. The sixth, and most recently established genus, *Testadenovirus*, consists of adenoviruses from testudinoid turtles [2], (https://talk.ictvonline.org/ictv-reports/ictv_9th_report/dsdna-viruses-2011/w/dsdna_viruses/93/adenoviridae, accessed on 10 October 2021). Host–virus codivergence as well as occasional host-switching events have been hypothesized to influence the evolution of adenoviruses [2,4]. It has been proposed that the mastadenoviruses, aviadenoviruses, atadenoviruses, siadenoviruses, ichtadenoviruses, and testadenoviruses coevolved with mammals, birds, reptiles, amphibians, fish, and turtles, respectively, suggesting continuous coevolution of adenoviruses with their vertebrate hosts [5]. On the other hand, the phylogenetic clustering of adenoviruses from unrelated host species within a genus, especially the detection of atadenoviruses (thought to coevolve with reptiles) in birds and ruminants, appears to reflect past host switching events [2,4,6].

Mongooses (family *Herpestidae*) are small terrestrial carnivorous mammals [7,8]. Due to their scavenging and invasive behavior, mongooses have been known to stray into human habitats and those of other animals, posing a risk as potential reservoirs of viral pathogens [7,9,10]. Mongooses have been identified as an important enzootic carrier of the rabies virus [10] and a potential reservoir of the hepatitis E virus [9]. Among other viruses, *Carnivore protoparvovirus 1*, cowpox virus, circoviruses, cycloviruses, feline panleukopenia virus, gemycircularviruses, picobirnavirus, and thogoto virus have been detected in mongooses, albeit from single studies [11,12,13,14,15,16,17]. To date, there is a lack of information on adenoviruses circulating in mongoose populations. Although a previous study conducted PCR-based screening on lung tissue samples (*n* = 28) from the Egyptian mongoose (*Herpestes ichneumon*) using canine adenovirus-1 and -2 specific primers, none of the animals tested positive for adenoviruses [13]. We report here the first-time detection and molecular characterization of adenoviruses from the small Indian mongoose (*Urva auropunctata*).

## 2. Materials and Methods

Between April–July 2017, 83 fecal samples were collected from the rectum and distal part of the colon of apparently healthy small Indian mongooses that were trapped, euthanized, and necropsied under sterile conditions for an intestinal microbiome study on the Caribbean island of St. Kitts [18]. Total DNA was extracted using the QIAamp Fast DNA Stool Mini Kit (Qiagen Sciences, Germantown, MD, USA) following the manufacturer’s instructions. The samples were screened for the presence of adenoviruses using a nested PCR assay (targeting a short region (~320 bp) of the viral DNA-dependent DNA polymerase (*pol*) gene) as described previously [19].

The PCR amplicons were purified using the Wizard^®^ SV Gel and PCR Clean-Up kit (Promega, Madison, WI, USA) following the instructions outlined by the manufacturer. Nucleotide (nt) sequences were obtained using the ABI Prism Big Dye Terminator Cycle Sequencing Ready Reaction Kit on an ABI 3730XL Genetic Analyzer (Applied Biosystems, Foster City, CA, USA). The standard BLASTN and BLASTP program (Basic Local Alignment Search Tool, www.ncbi.nlm.nih.gov/blast, accessed on 4 October 2021) was used to conduct a homology search for related nt and deduced amino acid (aa) sequences, respectively. Multiple alignment of the partial putative pol protein sequences (~90 aa) was performed using the CLUSTALW program (version ddbj, http://clustalw.ddbj.nig.ac.jp/, accessed on 4 October 2021) with default parameters. Phylogenetic analysis was carried out by the maximum likelihood (ML) method using the MEGA7 software [20], with the LG+I+G model of substitution and 1000 bootstrap replicates.

## 3. Results and Discussion

**Figure 1:**
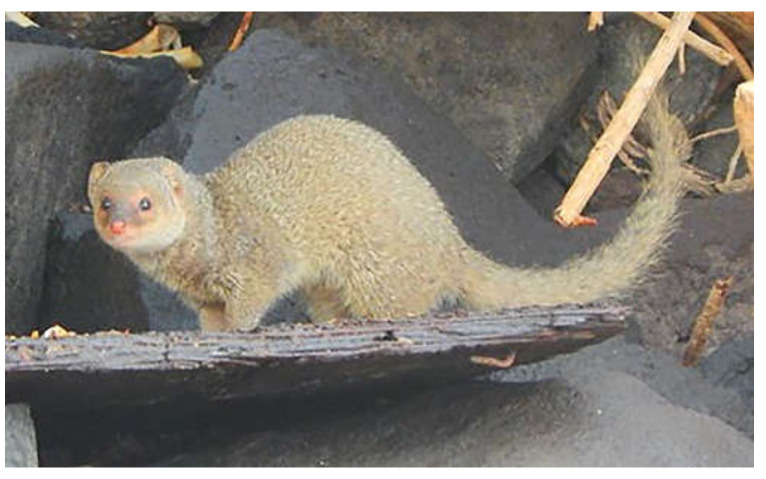
The small Indian mongoose (*Urva auropunctata*) on St. Kitts. The photograph of the small Indian mongoose was kindly provided by and used here with permission of Luis Cruz Martinez, Ross University School of Veterinary Medicine, St. Kitts and Nevis.

**Figure 2:**
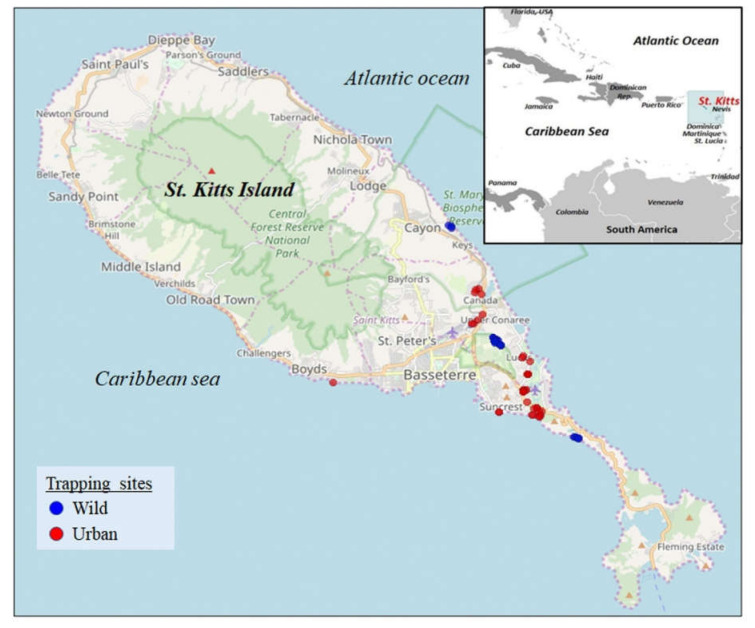
Geographical location of the Caribbean island of St. Kitts. The map was obtained from https://www.cia.gov/library/publications/the-world-factbook (Inset) (accessed on 1 April 2021). The mongoose trapping sites on St. Kitts. Blue and red indicate the trapping sites in the wild and urban habitats, respectively. The map was obtained from google maps (https://maps.google.com, accessed on 4 November 2019).

The island of St. Kitts (~69 square miles, human population of ~35,000) is home to a large population of the small Indian mongooses (~45,000) that thrive in wild and urban habitats (Figure 1 and Figure 2) [21]. Previously, we reported novel circoviruses, cycloviruses, and picobirnaviruses in the small Indian mongoose on St. Kitts [14,15]. These findings indicated that various viruses might be circulating in the island’s mongoose population, prompting us to screen the animals for adenoviruses. Since a previous study could not detect adenoviruses in mongooses using canine adenovirus-specific PCRs [13], we employed a broad-range nested PCR screening assay that has been used to amplify atadenoviruses, aviadenoviruses, mastadenoviruses, and siadenoviruses [19,22,23]. Seventeen (20.48%) of the 83 mongooses tested PCR positive for adenoviruses. All 17 PCR positive samples were sequenced for the partial *pol* gene (~300 nt, hereafter referred to as Mon sequences) and, using BLASTN analysis, shared maximum homology with adenoviruses from other host species.

**Figure 3:**
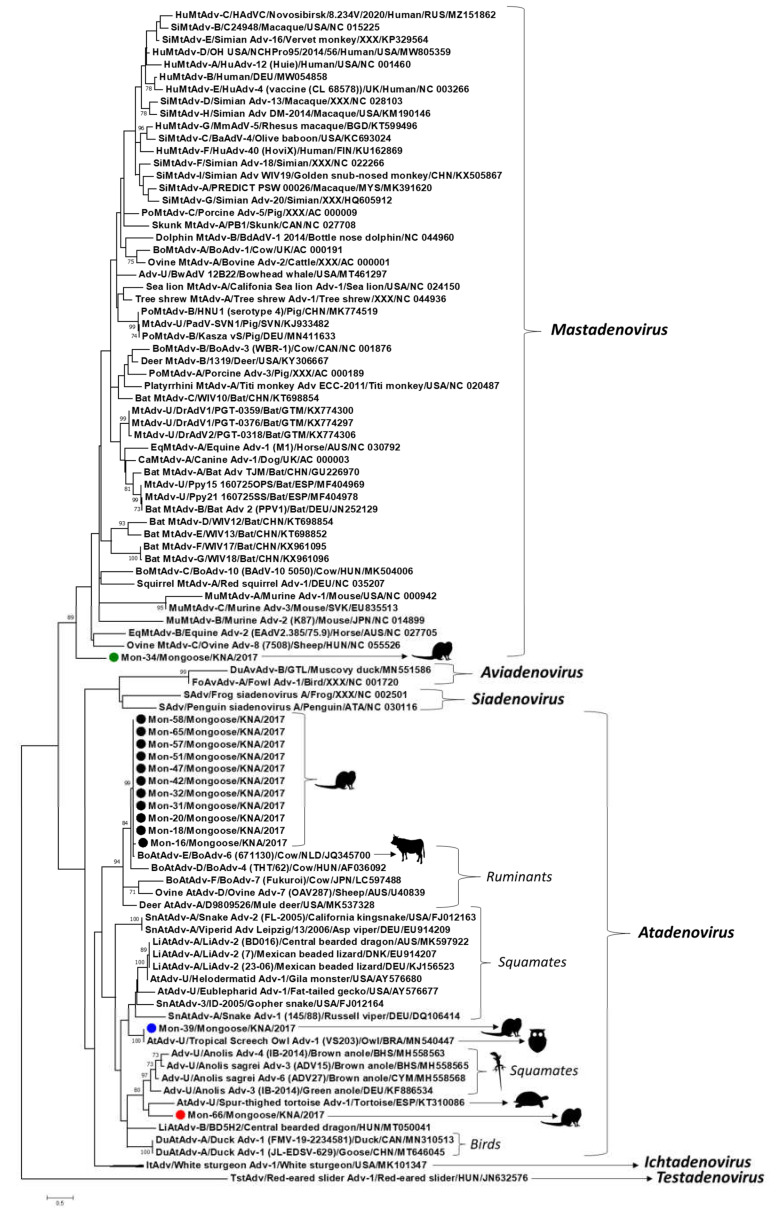
Phylogenetic analysis of the partial putative DNA-dependent DNA polymerase (pol) sequences (~90 amino acid (aa) residues) of the mongoose-associated adenoviruses with cognate adenovirus sequences from other host species. Mongoose-associated adenoviruses clustering within genus *Atadenovirus* are shown with black, blue, and red circles, respectively, whilst that related to mastadenoviruses is highlighted with a green circle. The tree was constructed by the maximum likelihood (ML) method using the MEGA7 software, with the LG+I+G model of substitution and 1000 bootstrap replicates. Unrooted calculation, the tree was rooted with red-eared slider adenovirus-1 (Genus *Testadenovirus*) for visualization. Scale bar, 0.5 substitutions per aa. Bootstrap values of <70 are not shown. Adv, *adenovirus*; Adv-U, *unclassified Adenoviridae*; AtAdv-U: *unclassified Atadenovirus*; BoAdv, *bovine Adenovirus*; BoAtAdv-E, *Bovine atadenovirus E*; BoMtAdv, *bovine Mastadenovirus*; CaMtAdv, *canine Mastadenovirus*; DrAdV, *Desmodus rotundus adenovirus*; DuAtAdv, *duck Atadenovirus*; DuAvAdv, *duck Aviadenovirus*; EqMtAdv, *equine Mastadenovirus*; FoAvAdv, *fowl Aviadenovirus*; HuMtAdv, *human Mastadenovirus*; ItAdv, *Ichtadenovirus*; LiAdv-2, *lizard adenovirus 2*; LiAtAdv-A, *Lizard atadenovirus A*; MtAdv-U, *unclassified Mastadenovirus*; MuMtAdv, *murine Mastadenovirus*; PoMtAdv-B, *Porcine mastadenovirus B*; SAdv, *Siadenovirus*; SiMtAdv, *simian Mastadenovirus*; SnAtAdv, *snake Atadenovirus*; TstAdv, *Testadenovirus*.

Fourteen of the 17 Mon sequences shared maximum homology with that of bovine adenovirus-6 (BoAdv-6) (species *Bovine atadenovirus E*) strain 671130 (GenBank accession number JQ345700, isolated from a latent infection of a calf testicular cell culture) [24]. However, sample Mon-33 yielded a low quality nt sequence, whilst high quality sequences of shorter lengths (<250 nt) were obtained for Mon-14 and Mon-52. As a result, Mon-14, Mon-33, and Mon-52 were excluded from further analysis. The remaining 11 of the 14 Mon sequences shared maximum nt and deduced aa identities of 98.6–100% and 97.5–100%, respectively, between themselves, and 98.3–99.6% and 97.0–98.9%, respectively, with that of BoAdv-6 (Table 1). By phylogenetic analysis, these Mon sequences formed a single group with BoAdv-6, within the ruminant atadenovirus cluster (Figure 3).

Phylogenetically, atadenoviruses from birds, reptiles, and ruminants constitute separate lineages within the genus *Atadenovirus* [2]. Since atadenoviruses have been proposed to coevolve with reptiles, it has been hypothesized that the avian and ruminant lineages might have emerged from ancient host-switching events involving reptiles [2,4]. To our knowledge, this is the first report on atadenoviruses from carnivorous mammals that were closely related to ruminant atadenoviruses. Bovine adenovirus-6 has been detected in bovine urine, manure, and ground water [25]. Considering the feeding habits of the small Indian mongoose [7], it might be possible that the BoAdv-6-like mongoose-associated atadenoviruses were of dietary origin, resulting from consumption of contaminated cow dung, or water. The detection of four of the BoAdv-6-like viruses from a trapping location that serves as grazing area to cattle supported this observation. On the other hand, the remaining 10 BoAdv-6-like viruses were detected from sampling sites where ruminants are usually not present, warranting further investigation into the exact origin of these mongoose-associated atadenovirus strains.

Mongoose-associated adenovirus Mon-39 was most closely related to the partial *pol* sequence of an atadenovirus from a tropical screech owl (nt and deduced aa identities of 100%) [26], followed by atadenoviruses from reptiles (nt and deduced aa identities of 72.7% and 78.9–80%, respectively) (Figure 3, Table 1). Mongoose-associated adenovirus Mon-66 shared maximum nt and deduced aa identities of 69% and 71.4% with that of atadenoviruses from a spur-thighed tortoise and a brown anole lizard, respectively (Table 1) [27,28]. Phylogenetically, Mon-66 formed a distinct branch, near the tortoise atadenovirus, within a cluster that also consisted of atadenoviruses from anole lizards (Figure 3). Mon-39 and Mon-66 shared deduced aa identities of 66.6% between themselves, and 61.0–65.8% and 55.5–60.2%, respectively, with the BoAdv-6-like mongoose-associated atadenoviruses. It has been proposed that the atadenovirus from the tropical screech owl might have originated from a preyed reptile [26]. On the other hand, the source of the chelonian atadenovirus, associated with an infection of the digestive tract in the tortoise, could not be determined [28]. The tropical island of St. Kitts has a sizeable population of lizards (anole lizards and geckos) that are preyed upon by the small Indian mongoose (https://www.cbd.int/doc/world/kn/kn-nbsap-01-p5-en.pdf, accessed on 12 October 2021). Considering the phylogenetic clustering patterns (Figure 3) and that Mon-39 and Mon-66 were detected in fecal samples, we suspect that these mongoose-associated atadenoviruses might have originated from consumed lizards.

Although the small Indian mongoose is a carnivorous mammal, only a single mongoose-associated adenovirus, Mon-34, was related to the genus *Mastadenovirus* (Figure 3, Table 1). Mon-34 shared maximum nt and deduced aa identities of 72.0% and 72.6% with *Desmodus rotundus* adenovirus-1 (isolate PGT-0359, from a bat in Guatemala) and bowhead whale adenovirus strain BwAdV 12B22 (from a bowhead whale in Alaska, USA), respectively (Table 1) [29,30]. Phylogenetically, Mon-34 formed an isolated branch, distinct from other mastadenoviruses, within the genus *Mastadenovirus* (Figure 3). Based on sequence identities and phylogenetic analysis of partial pol, Mon-34 appeared to represent a separate lineage of mastadenoviruses that might be circulating in mongooses (Figure 3, Table 1). However, analysis of the complete genomes of several mongoose-associated mastadenoviruses might be required to corroborate this hypothesis.

Taken together, our findings revealed the wide distribution and genetic diversity of adenoviruses in the small Indian mongoose population on St. Kitts. Since the present study was based on fecal samples from apparently healthy mongooses, we could not determine if the mongoose-associated adenoviruses actually infected the host. On the other hand, the phylogenetic clustering patterns of the mongoose-associated atadenoviruses pointed more towards a dietary origin of these viruses (Figure 3). The partial pol sequences of mongoose-associated adenovirus Mon-34, Mon-39 (and that of tropical screech owl adenovirus 1), and Mon-66 shared low deduced aa identities with those of other adenoviruses (Table 1), and based on phylogenetic distance (≥29.6%, ≥23.2%, and ≥28.0%, respectively), might represent novel adenoviruses (Figure 3). However, the present study was based on short pol sequences (~90 aa), which is insufficient to obtain conclusive information on the evolution of the mongoose-associated adenoviruses. Although attempts were made to amplify the hexon gene of the mongoose-associated adenoviruses using broad-spectrum primers as described previously [31], none of the samples yielded positive results. Future studies based on the detection of adenovirus DNA in tissue samples and determination of the complete genomes of novel adenoviruses (that are difficult to amplify by conventional PCRs) using next generation sequencing technologies might be required to gain a proper understanding of the evolution and pathogenic potential of adenoviruses in mongoose populations. To our knowledge, this is the first report on detection and molecular characterization of adenoviruses from the mongoose.

## Figures and Tables

**Table 1:** Maximum/significant nucleotide (nt) and deduced amino acid (aa) identities (%) shared by the partial (~300 nt and ~90 aa, respectively) DNA-dependent DNA polymerase (pol) encoding sequences of the mongoose-associated adenoviruses with those from other host species.

Mongoose-Associated Adenovirus	GenBank Accession Number/s	Genus ^1^	Maximum/Significant Identity with Cognate Adenovirus Sequences (Species ^2^/Organism (Isolate/Strain)/Host/Country/GenBank Accession Number) from Other Host Species	
			Nucleotide Sequence Identity (%) ^3^	Deduced aa Sequence Identity (%) ^4^
Mon-16, Mon-18, Mon-20, Mon-31, Mon-32, Mon-42, Mon-47, Mon-51, Mon-57, Mon-58, Mon-65	OK381854–OK381858, OK381861–OK381863, OK381865–OK381867	*Atadenovirus*	98.3–99.6% with *Bovine atadenovirus E/* *Bovine adenovirus-6 (671130)/Cow/Netherlands/JQ345700*	97.0–98.9% with *Bovine atadenovirus E/* *Bovine adenovirus-6 (671130)/Cow/Netherlands/JQ345700*
Mon-34	OK381859	*Mastadenovirus*	72.0% with *Unclassified Mastadenovirus/Desmodus rotundus Adenovirus-1 (DrAdV1/PGT-0359)/Bat/Guatemala/>KX774300* 70.5% with *Unclassified Mastadenovirus/Desmodus rotundus Adenovirus-2 (DrAdV2/PGT-0318)/Bat/Guatemala/KX774306*	72.6% with *Unclassified Adenoviridae/Bowhead whale adenovirus (BwAdV 12B22)/Bowhead whale/USA/MT461297* *72.3% with Unclassified Mastadenovirus/Desmodus rotundus Adenovirus-1 (DrAdV1/PGT-0359)/Bat/Guatemala/KX774300 and Unclassified Mastadenovirus/Desmodus rotundus Adenovirus-2 (DrAdV2/PGT-0318)/Bat/Guatemala/KX774306*
Mon-39	OK381860	*Atadenovirus*	100% with *Unclassified Atadenovirus/Tropical screech owl adenovirus-1 (VS203)/Owl/* *Brazil/MN540447* 72.7% with *Lizard Atadenovirus-A/Lizard * *adenovirus-2 (23-06)/Mexican beaded lizard/* *Germany/KJ156523*	100% with *Unclassified Atadenovirus/Tropical screech owl adenovirus-1 (VS203)/Owl/* *Brazil/MN540447* 80.0% with *Unclassified Atadenovirus/* *Helodermatid adenovirus-1/Gila monster/USA/AY576680* 78.9% with *Lizard Atadenovirus-A/Lizard adenovirus-2 (23-06)/Mexican beaded lizard/* *Germany/KJ156523*
Mon-66	OK381868	*Atadenovirus*	69.0% with *Unclassified Atadenovirus/* *Spur-thighed tortoise adenovirus-1 (6211)* */Tortoise/Spain/KT310086* ^5^ 68.7% with *Unclassified Adenoviridae/* *Anolis sagrei adenovirus-3 (ADV15)/Brown anole/Bahamas/MH558565* 68.2% with *Unclassified Adenoviridae/* *Anolis sagrei adenovirus-6 (ADV27)/Brown anole/Cayman Islands/MH558568*	71.4% with *Unclassified Adenoviridae/Anolis sagrei adenovirus-6 (ADV27)/Brown anole/Cayman Islands/MH558568* 70.0% with *Snake Atadenovirus-A/Snake adenovirus-2 (FL-2005)/California kingsnake/USA/FJ012163* 70.0% with *Unclassified Atadenovirus/* *Spur-thighed tortoise adenovirus-1 (6211)/* *Tortoise/Spain/KT310086*

^1^ Based on sequence identities and phylogenetic analysis (Figure 3) of the partial *pol* sequences of the mongoose-associated adenoviruses. ^2^ As described in the NCBI Taxonomy Browser (https://www.ncbi.nlm.nih.gov/Taxonomy/Browser/wwwtax.cgi?id=559881, accessed on 5 October 2021). ^3, 4^ Based on BLASTN ^3^ and BLASTP ^4^ analysis (Basic Local Alignment Search Tool, www.ncbi.nlm.nih.gov/blast, accessed on 4 October 2021). ^5^ Determined using the CLUSTALW program (version ddbj, http://clustalw.ddbj.nig.ac.jp/, accessed on 4 October 2021) with default parameters.

## Data Availability

Not applicable.
